# Emerging antiviral drugs and immunotherapy for chronic hepatitis B and associated liver disease

**DOI:** 10.3389/fimmu.2026.1806527

**Published:** 2026-05-18

**Authors:** Chunting Zhu, Jie Yang, Yu Sheng, Min Fang

**Affiliations:** 1 Department of Nursing, the Fourth Affiliated Hospital of School of Medicine, and International School of Medicine, International Institutes of Medicine, Zhejiang University, Yiwu, China; 2 Department of Infectious Disease, the Fourth Affiliated Hospital of School of Medicine, and International School of Medicine, International Institutes of Medicine, Zhejiang University, Yiwu, China

**Keywords:** antiviral drug, chronic hepatitis B, HBsAg clearance, HBV, immune checkpoint inhibitor, immunotherapy, liver disease, vaccine

## Abstract

Chronic hepatitis B virus (HBV) infection is a major global cause of chronic hepatitis, cirrhosis, and hepatocellular carcinoma. Despite current antiviral therapies, functional cure remains elusive due to persistent HBsAg expression and immune dysfunction. Recent therapeutic advances include direct-acting antivirals, such as entry inhibitors, siRNAs, antisense oligonucleotides, capsid assembly modulators, and HBsAg release blockers, and immunomodulatory approaches like therapeutic vaccines, Toll-like receptor (TLR) agonists, and immune checkpoint inhibitors. Combination regimens that integrate viral suppression with immune restoration offer the most promising route toward sustained HBV control. Nevertheless, challenges such as limited seroconversion, viral rebound, and safety concerns persist. This review systematically summarizes recent progress in antiviral and immunotherapeutic strategies for chronic HBV infection associated hepatitis, highlights their underlying mechanisms and clinical efficacy, and underscores the future value of rational combination therapies in achieving functional cure and reducing hepatitis B burden.

## 1 Introduction

Hepatitis B virus (HBV) infection represents a major global public health concern (1, 2). Currently, suppressing HBV replication, alleviating or preventing hepatic fibrosis, and reducing the incidence of cirrhosis and HCC represent the principal therapeutic goals for patients with chronic HBV infection worldwide (3, 4). Serum hepatitis B surface antigen (HBsAg) levels are considered one of the most important indicators for evaluating disease progression and functional cure in chronic HBV infection. HBsAg levels below 100 IU/mL exhibit a significantly reduced incidence of liver fibrosis, whereas individuals with hepatitis B e antigen (HBeAg) positivity, HBV DNA levels exceeding 10^6^ IU/mL, and HBsAg levels above 1,000 IU/mL show an increased risk of developing HCC (5, 6). Compared with patients who are HBeAg-positive and have high HBsAg levels, those who are HBeAg-negative and have lower HBsAg concentrations are more likely to achieve HBsAg clearance (7). Currently, nucleos(t)ide analogues (NAs) and pegylated interferon-α (PEG-IFN-α) are the most commonly used antiviral agents for the treatment of HBV infection (8, 9). However, regardless of whether these agents are administered as monotherapy or combination therapy, the overall rate of HBsAg clearance remains relatively low (10–13). Therefore, the development of novel therapeutic strategies to further enhance HBsAg clearance and facilitate the achievement of functional cure in HBV infection remains a critical unmet need in the field of hepatitis B research.

## 2 Novel therapeutic strategies for chronic HBV infection associated hepatitis

Emerging evidence indicates that covalently closed circular DNA (cccDNA), the integration and expression of HBV DNA, and dysregulation of host immune responses are the principal factors contributing to the persistence of HBV infection (14). Therefore, strategies aimed at suppressing or eliminating cccDNA and its viral products, including HBsAg, HBeAg, and hepatitis B core-related antigen (HBcrAg), as well as therapeutic approaches designed to enhance virus-specific cellular immune responses, are all expected to facilitate HBV clearance (15, 16). Direct-acting antiviral agents include entry inhibitors, small interfering RNA (siRNA), antisense oligonucleotides (ASOs), HBsAg inhibitors, and capsid assembly modulators (17, 18). Indirect-acting antiviral strategies include therapeutic vaccines, immune checkpoint inhibitors, Toll-like receptor (TLR) agonists, and immunomodulatory agents. These therapies aim to enhance antiviral immune responses by improving immune cell function, thereby promoting viral clearance (19).

### 2.1 TLRs-related drug

Entry inhibitors primarily target the sodium taurocholate cotransporting polypeptide (NTCP), the functional receptor mediating HBV entry into hepatocytes. These agents exert antiviral effects by competitively binding the preS1 domain of the HBV large envelope (L) protein, thereby blocking viral attachment to NTCP (20). Currently, Myrcludex B is the most extensively investigated. Its clinical application has focused largely on hepatitis D virus (HDV) infection and HBV/HDV coinfection. Clinical studies demonstrate that Myrcludex B effectively suppresses HDV RNA levels and promotes normalization of alanine transaminase (ALT). Compared with PEG-IFN-α, it exhibits a more favorable safety profile, with transient transaminase elevation as the most common adverse event and no reported treatment-related mortality (21, 22). In addition, NTCP contributes to the regulation of HBV transcription and replication. NTCP upregulation enhances viral replication, whereas this effect is attenuated by NTCP inhibitors such as Myrcludex B (23, 24). In parallel, monoclonal antibodies targeting NTCP, including HH-006, HH-003, and Burbiralimab have entered clinical development, and their safety and efficacy warrant further investigation. Moreover, cyclopeptide derivatives SCY995 and SCY450 competitively bind NTCP via the preS1 region, thereby inhibiting HBV infection without disrupting bile acid transport (25–27).

### 2.2 Small interfering RNA agent

siRNA comprises a class of double-stranded short RNA molecules that exerts antiviral activity primarily by directing the degradation of HBV transcripts, thereby suppressing the production of viral proteins, including HBeAg and HBsAg, as well as viral particle assembly (28, 29). Several siRNA-based agents have entered clinical evaluation. For example, VIR-2218 and JNJ-73763989, both targeting the HBx open reading frame, have been shown to rapidly lower HBsAg levels (30, 31). Relative to monotherapy, combination regimens with nucleos(t)ide analogues and capsid assembly modulators achieved greater HBsAg reductions, although HBsAg clearance remained uncommon. The antiviral efficacy of siRNA appears to be dose- and time-dependent, yet largely independent of baseline HBsAg levels and HBeAg status. Notably, HBsAg rebound has been documented after siRNA treatment and is associated with higher dosing, longer treatment duration, and delayed onset of rebound (32–34). In patients with suboptimal responses to NAs, the combination of JNJ-73763989 with capsid assembly inhibitors such as JNJ-6379 has shown encouraging activity, with approximately 81.5% of patients achieving HBsAg reduction (31). In contrast to JNJ-73763989 and VIR-2218, RG6346 targets mRNA regions outside the HBx coding sequence. Clinical studies showed that HBsAg reduction with RG6346 was not dose-dependent, and that this agent was associated with delayed rebound and lower HBsAg recurrence than JNJ-73763989 and VIR-2218 (35). Together, these findings indicate that siRNA target-site selection critically shapes therapeutic efficacy, and that HBx-targeting transcripts may be suboptimal for suppressing HBsAg synthesis. Overall, although siRNA-based therapy effectively reduces HBsAg, its efficacy remains dose- and time-dependent, and only a minority of patients achieve HBsAg seroconversion. Combination with immunomodulatory strategies may therefore be necessary to enhance viral clearance. Consistently, recent preliminary clinical data indicate that siRNA combined with immunotherapeutic approaches, including PEG-IFN, TLR agonists, and therapeutic vaccines, yields greater HBsAg decline, higher HBsAg seroconversion rates, and more durable suppression of rebound (36, 37).

Despite the generally favorable safety profile reported in earlier studies, safety liabilities associated with specific siRNA agents have led to discontinuation of clinical development. For example, ARC-520 showed promising antiviral activity in phase Ib/II trials, with marked reductions in HBsAg after treatment. Sustained HBsAg declines lasting nearly three months were observed, and among six patients who underwent long-term follow-up after sequential ARC-520 and NAs therapy, one achieved HBsAg seroconversion. Nevertheless, clinical development of ARC-520 was terminated because of fatal toxicity linked to the delivery carrier used in the trial (38–40). These adverse events were attributed to the EX1 delivery vector (41). These findings highlight that chemical modifications, delivery platforms, and target specificity may contribute to adverse effects associated with siRNA therapy. Therefore, future clinical studies need to carefully monitor treatment-related adverse events and further optimize safety evaluation systems.

### 2.3 Antisense oligonucleotide therapy

Antisense oligonucleotides (ASOs) are single-stranded RNA molecules that suppress viral protein production through complementary base pairing with target HBV mRNA or pre-mRNA. Reported ASO agents include RO7062931, bepirovirsen and GSK3389404. In a phase I trial in patients with chronic hepatitis B (CHB), RO7062931 induced dose-dependent reductions in HBsAg and rebound kinetics that were independent of baseline HBeAg status. Adverse events were not associated with treatment severity, although ALT elevations occurred more frequently at higher doses (42). Phase II trials evaluating the safety and antiviral activity of bepirovirsen showed that, in addition to ALT and AST elevations, increases in C-reactive protein emerged early during treatment. Within four weeks, HBsAg reduction was dose-dependent, and transient HBsAg seroconversion was observed in four patients, although HBsAg reappeared during follow-up (43, 44). In phase I studies, GSK3389404 also induced dose-dependent reductions in HBsAg, however, no HBsAg seroconversion was detected (45).

Similar to siRNA therapies, discontinuation of ASO treatment may result in HBsAg rebound in certain patients, suggesting that direct antiviral therapy alone may be insufficient to achieve functional cure (44). The combination therapy incorporating immunomodulatory strategies is expected to represent a future direction in the treatment of chronic HBV infection (46, 47). Notably, ASO therapeutics have not shown clear superiority over siRNA in suppressing HBV RNA, HBcrAg or HBeAg, except potentially in HBeAg-positive populations, in whom targeting mRNA encoding HBeAg may represent a key therapeutic determinant, although the underlying mechanisms remain unclear (36, 48). By contrast, ASOs (bepirovirsen) may hold greater promise for HBsAg clearance than siRNA-based agents (10). Preclinical studies further suggest that Bepirovirsen may enhance antiviral efficacy by activating Toll-like receptor signaling pathways and augmenting innate immune responses. Additionally, whereas siRNA-mediated mRNA degradation may lose efficacy against shared target regions within integrated HBV transcripts, ASOs primarily act through direct pairing with otherwise silent mRNA sequences, thereby reducing transcription from integrated HBV DNA (40) These mechanistic differences may partially explain the variations in therapeutic efficacy between ASO and siRNA therapies.

### 2.4 HBsAg inhibitors

Unlike siRNA and ASO approaches, which directly suppress HBsAg synthesis, HBsAg inhibitors primarily exert antiviral activity by preventing HBsAg release from infected hepatocytes. REP 2139 and REP 2165 are representative nucleic acid polymers (NAPs) that have shown promising therapeutic activity in chronic HBV infection (45, 49). an open-label, randomized, controlled phase II trial assessed REP 2165 or REP 2139 in combination with PEG-IFN-α and tenofovir disoproxil fumarate in patients with chronic hepatitis B (CHB), demonstrating that triple NAP-based therapy reduced HBsAg to below the limit of detection in all treated patients (50). Beyond NAPs, other HBsAg-targeting agents, including dihydroquinolizinone derivatives GST-HG121 and GST-HG131, as well as the small-molecule drug BJT-574, are currently under investigation for their efficacy and safety and may provide new therapeutic strategies for HBV infection (51). The combined therapeutic effects of PEG-IFN and NAPs may result in circulating HBsAg reductions, which facilitate reactivation of host immune responses and enhance viral clearance (15, 52). Importantly, profound reductions in circulating HBsAg may also relieve persistent antigenic stimulation, thereby promoting the restoration of HBV-specific T-cell responses, including improved proliferative capacity and antiviral effector function (53, 54). Moreover, NAPs have been suggested to exert hepatoprotective effects by interacting with inflammatory cytokines, inhibiting receptor activation, and mitigating ALT elevation-associated hepatic injury (15, 53). However, the precise mechanisms by which NAPs suppress hepatic inflammatory activation remain to be fully elucidated.

### 2.5 Capsid assembly modulators

**Figure 1:**
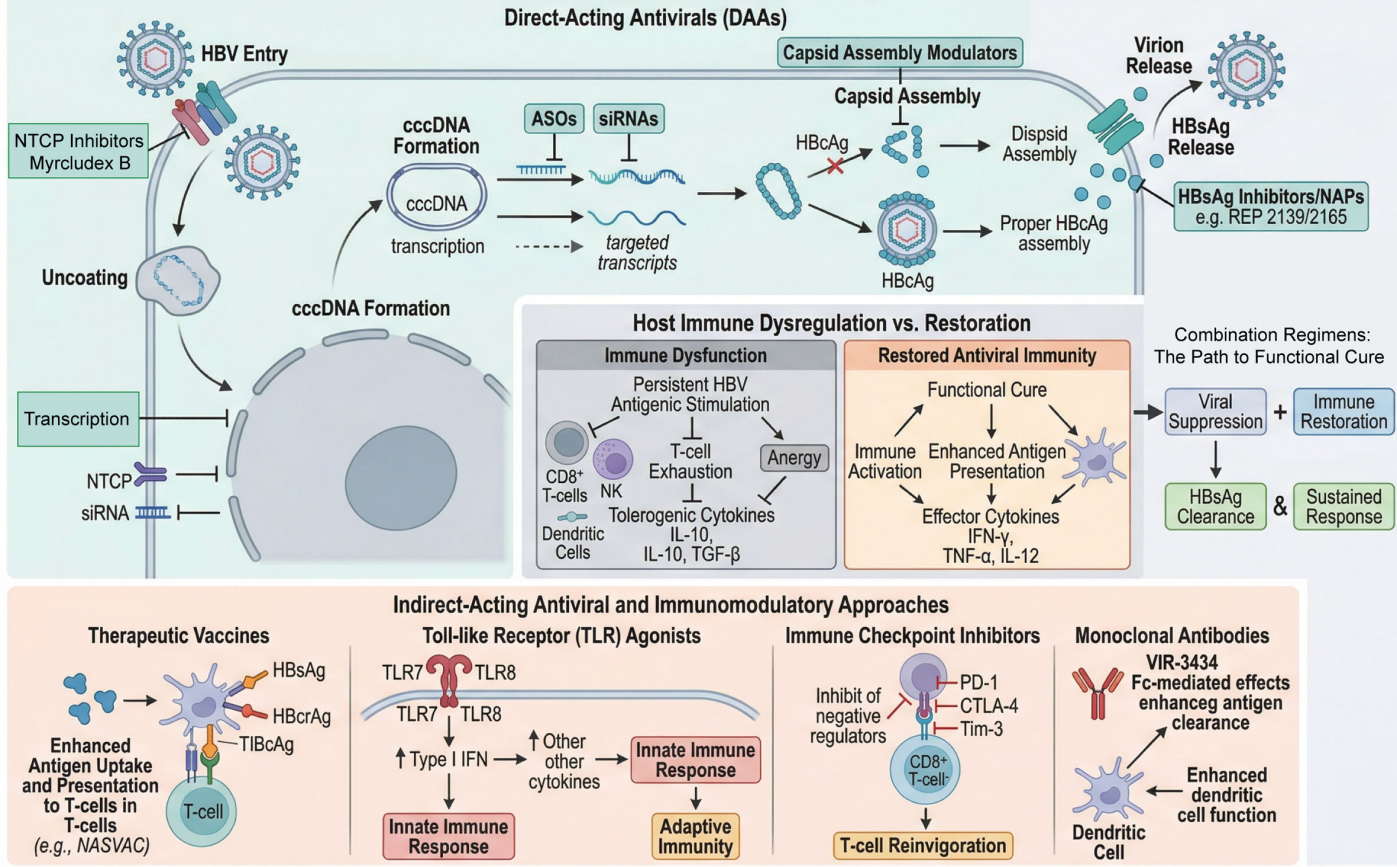
Emerging antiviral drugs and immunotherapy for Chronic Hepatitis B.

Following HBV RNA transcription and translation, viral nucleocapsid assembly proceeds to generate mature virions. A fraction of these particles is secreted to infect adjacent hepatocytes, whereas others are recycled to the nucleus and converted into cccDNA, thereby sustaining persistent HBV replication (55). Thus, disruption of nucleocapsid assembly or promotion of capsid protein degradation can effectively impede viral replication. According to their mechanisms of action, capsid assembly modulators are broadly categorized into three classes (56). The first class disrupts canonical capsid assembly while promoting core protein degradation, and includes TQA3605, QL-007, and GLS4 (57). The second induces the formation of empty capsids containing pregenomic RNA (pgRNA), with representative agents including EDP-514, ZM-H1505R, and ABI-4334 (58). The third category binds to preassembled capsid proteins and disrupts capsid structural integrity; ABI-H0731 is representative of this class (59). Clinical investigations of capsid assembly modulators have demonstrated that these agents effectively suppress HBV DNA and HBV RNA levels; however, viral rebound has been observed following treatment discontinuation (60). Moreover, these agents exert minimal effects on HBsAg levels, and most clinical studies have not demonstrated significant reductions in HBsAg (61). Therefore, capsid assembly modulators alone may be insufficient to reduce the risk of cirrhosis and HCC, highlighting the need for combination therapy with other antiviral or immunomodulatory agents to enhance HBsAg suppression (62). Recent preliminary clinical studies reported that combination therapy involving capsid assembly modulators, NAs, and PEG-IFN-α effectively reduces HBsAg levels (63) (**Figure 1**).

## 3 Indirect-acting antiviral agents for chronic HBV infection associated hepatitis

### 3.1 Therapeutic vaccines

Like prophylactic vaccines, therapeutic vaccines employ viral proteins or peptides as immunogens to activate host immune responses, thereby inducing the production of long-lasting antibodies and memory immune cells (64). NASVAC remains one of the most extensively investigated candidates, which is formulated by combining HBcrAg expressed in *Escherichia coli* with HBsAg virus-like particles expressed in *Pichia pastoris*. Unlike conventional antiviral therapies, NASVAC primarily enhances host immune responses (65, 66). In Phase I and phase III clinical trials, NASVAC exhibits favorable safety profiles, with most adverse events limited to localized nasal reactions and no evidence of chronic mucosal inflammatory responses. Approximately 90% of patients developed seroconversion of anti-HBc and anti-HBs antibodies, accompanied by significant reductions in HBV DNA levels and normalization of ALT in some patients (67, 68). Long-term follow-up studies revealed that certain patients maintained detectable anti-HBs and anti-HBc antibodies, with sustained reductions in HBV DNA and ALT levels and no occurrence of cirrhosis or HCC events (69, 70). Subsequent investigations further optimized NASVAC by incorporating carboxyl vinyl polymer (CVP), a mucoadhesive material commonly used in nasal drug delivery systems, along with a specialized intranasal delivery device. Preclinical studies have shown that, compared with conventional nasal delivery systems, CVP enhances the retention time of NASVAC in the nasal cavity, thereby improving its capacity to induce antiviral immune responses (68) (71).

In addition to monotherapy, the feasibility of combining therapeutic vaccines with antiviral agents or immunotherapy is being actively explored. In patients previously treated with NAs, BRI-179 administered with or without PEG-IFN-α showed a favorable safety profile, and seroconversion of anti-HBs and anti-HBc antibodies was observed in more than 30% of cases. Notably, however, anti-preS1 and anti-preS2 antibody responses were detected only in the cohorts receiving PEG-IFN-α-based combination therapy (72, 73). Likewise, GS-4774 combined with tenofovir produced an inhibitory effect on HBsAg comparable to that achieved with tenofovir alone (74). Subgroup analyses nevertheless suggested that patients with elevated ALT levels and HBeAg positivity derived greater reductions in HBsAg than other patient subsets (75). In patients with HBeAg negativity and low baseline HBsAg levels, GS-4774 combined with the PD-1 inhibitor nivolumab not only resulted in reductions in HBsAg levels but also achieved HBsAg clearance in one patient (76).

### 3.2 Toll-like receptor agonists

TLR agonists enhance host immunity by inducing antiviral and immunoregulatory cytokines, thereby reactivating and restoring exhausted or senescent innate and adaptive immune responses and improving antiviral immune defense (77). TLR agonists primarily target TLR7 and TLR8 (78). In clinical, TLR agonist therapy in patients with chronic hepatitis B (CHB) exhibits acceptable safety and tolerability, with headache, nausea, and fatigue representing the most frequently observed adverse events, and no treatment-related serious adverse events reported (79, 80). Peripheral immune profiling revealed that TLR agonists induced transient, dose-dependent elevations in circulating cytokines, including TNF-α, interferon gamma-induced protein 10 (IP-10), interleukin-1 receptor antagonist (IL-1Ra), monocyte chemoattractant protein-1 (MCP-1), interleukin-12p40, interferons, CXCL8, and CCL20, thereby promoting the redistribution of circulating immune cell subsets (81). Although TLR agonist therapy has not yet demonstrated robust direct antiviral suppression, the observed activation of immune responses provides a compelling rationale for continued investigation of therapeutic vaccine–based cure strategies. Furthermore, combining TLR agonists with other therapeutic modalities, extending treatment duration, and prolonging follow-up periods may enhance the likelihood of achieving functional cure endpoints.

### 3.3 Immune checkpoint inhibitors and monoclonal antibodies

Immune checkpoint inhibitors exert antiviral effects by blocking inhibitory signaling pathways, thereby reversing immune cell exhaustion and functional impairment (82). Although current studies on immune checkpoint inhibitors for chronic HBV infection remain limited, this therapeutic strategy demonstrates considerable potential. For instance, clinical investigations involving nivolumab combined with therapeutic vaccination have observed reductions and clearance of HBsAg. Similarly, the PD-1 antibody ASC22 has shown dose-dependent reductions in HBsAg levels and decreases in HBV viral load in patients with CHB (15, 83). However, immune checkpoint inhibitor therapy may increase the risk of HBV reactivation and hepatitis flares. Furthermore, in CHB patients receiving such treatments, baseline HBsAg and HBV DNA levels are often relatively low, and therapeutic efficacy in patients with high viral loads requires further investigation (84, 85).

VIR-3434 is a subcutaneously administered monoclonal antibody that enhances antigen presentation by dendritic cells through Fc-mediated interactions with specific Fcγ receptors, thereby strengthening T-cell immune responses (86, 87). VIR-3434 monotherapy has demonstrated remarkable efficacy in suppressing HBsAg levels (88). Additionally, treatment cohorts receiving VIR-2218 combined with VIR-3434, with or without PEG-IFN therapy, demonstrated reductions of HBsAg. During post-treatment follow-up, HBsAg reappearance was detected in the VIR-3434 and VIR-2218 combination group, whereas sustained HBsAg clearance was observed in the triple-therapy cohort (30). Immunomodulatory therapy may be more advantageous in patients with low baseline HBsAg levels, whereas patients with high HBsAg levels face a higher risk of HBV reactivation. Notably, VIR-2218 can rapidly reduce HBsAg levels, enabling patients to derive greater benefit from VIR-3434-mediated immunomodulation, while PEG-IFN contributes to long-term viral suppression and immune activation, thereby maximizing antiviral efficacy. This principle may also be applicable to other combination therapeutic strategies (**Table 1**).

**Table 1:** Emerging antiviral and immunotherapy strategies for chronic hepatitis B.

Treatment strategy	Representative agents	Mechanism	Principal strength	Clinical value
siRNA therapy	VIR-2218, JNJ-73763989, RG6346, ARC-520	Degrade HBV transcripts, thereby reducing viral protein synthesis and particle production	Rapid HBsAg reduction; strong capacity to debulk viral antigen burden	Antigen-lowering backbone to sensitize patients to immune-restorative therapies
HBsAg inhibitors/NAPs	REP 2139, REP 2165, GST-HG121, GST-HG131, BJT-574	Inhibit HBsAg release rather than directly blocking its synthesis	Profound reduction in circulating HBsAg and relief of chronic antigenic pressure	Potent antigen-depletion strategy to facilitate immune restoration
Therapeutic vaccine	NASVAC, BRII-179, GS-4774	Restore or amplify HBV-specific adaptive immunity	Immunologically rational, generally well tolerated, and potentially synergistic with antigen-lowering agents	Immune reconstitution module after viral antigen burden has been reduced
TLR agonist	TLR7 and TLR8 agonists	Induce antiviral cytokines and activate innate–adaptive immune crosstalk	Reinvigorates antiviral immunity and promotes immune-cell redistribution	Immune-priming component for vaccine-based or multimodal cure strategies
Immune checkpoint inhibitor	Nivolumab, ASC22	Reverse immune exhaustion through blockade of inhibitory pathways such as PD-1 signaling	May restore HBV-specific T-cell function and reduce HBsAg in selected settings	Precision immunotherapy for carefully selected low-antigen or preconditioned patients
Immunomodulatory monoclonal antibody	VIR-3434	Enhances dendritic-cell antigen presentation through Fcγ receptor engagement and promotes T-cell activation	Mechanistically attractive bridge between antigen reduction and immune restoration	Combination immunotherapy, particularly with siRNA and/or PEG-IFN

## 4 Conclusion

With the continued expansion of the antiviral armamentarium, therapeutic strategies for chronic hepatitis B virus (HBV)-associated hepatitis are increasingly being reoriented toward the achievement of functional cure and, ultimately, complete clinical cure. Relative to conventional antiviral regimens, these emerging agents offer several notable advantages, yet each class remains constrained by specific mechanistic and clinical limitations. Small interfering RNAs (siRNAs), antisense oligonucleotides (ASOs), HBsAg inhibitors and capsid assembly modulators, for example, can induce rapid declines in circulating HBsAg, thereby providing an important foundation for therapeutic intervention; however, the magnitude and durability of their antiviral effects are frequently influenced by dose intensity and treatment duration. Likewise, entry inhibitors can efficiently block hepatocyte infection, but their interference with physiological bile acid transport raises potential concerns regarding tolerability and off-target effects.

By contrast, therapeutic vaccines and immunomodulatory agents appear to exert greater benefit in patients with relatively low viral burdens, whereas their efficacy is often diminished in the setting of high-level viraemia, where profound immune dysfunction and persistent antigen exposure remain major barriers to response. These considerations collectively underscore that no single therapeutic modality is likely to be sufficient for broad and durable HBV control. Rather, combination therapy is poised to become the central paradigm in the management of chronic hepatitis B. The rational integration of agents with distinct antiviral targets and complementary immunological effects may enable deeper suppression of viral replication, more effective reduction of viral antigens and more robust restoration of HBV-specific immune function. Continued interdisciplinary efforts spanning basic research, translational investigation and clinical practice will be crucial for overcoming the remaining barriers to cure and for accelerating the transition from viral suppression to functional cure, and ultimately to complete clinical cure, in chronic HBV-associated hepatitis.
